# The Role of the Appendix Testis in Normal Testicular Descent: Is There a Connection?

**DOI:** 10.1155/2018/3078031

**Published:** 2018-04-23

**Authors:** Zlatan Zvizdic, Dragana Zivkovic, Jasmin Sabanovic, Emir Milisic

**Affiliations:** ^1^Clinic of Pediatric Surgery, Clinical Center University of Sarajevo, Sarajevo, Bosnia and Herzegovina; ^2^Medical Faculty, University of Sarajevo, Sarajevo, Bosnia and Herzegovina; ^3^Department of Urology, Institute for Children and Youth Health Care of Vojvodina, Novi Sad, Serbia

## Abstract

**Objective:**

The presence of testicular appendices was prospectively evaluated in 89 boys with 96 undescended testes who underwent orchidopexy over the period of 4 years.

**Results:**

The patients were divided into two groups. Group A included 42 boys with 49 undescended testes positioned close to the internal inguinal ring, and Group B included 47 boys with 47 undescended testes close to the external inguinal ring. The incidence of appendix testis (AT) in Group A was 57.1% (28 in 49) and 78.7% (37 in 47) in Group B. The results of our study showed significantly decreased incidence of testicular appendices in undescended testes positioned close to the internal inguinal ring compared with undescended testes positioned close to the external inguinal ring (*p* < 0.05).

**Conclusion:**

AT may play a role in normal testicular descent and the undescended testis positioned close to the external inguinal ring can be considered as a separate entity of the true congenital undescended testis.

## 1. Introduction

Normal testicular descent (TD) is a complex, multistage process that involves coordinated action of various anatomical structures, hormones, environment, and genetic factors [1]. It is well known that TD occurs in two distinct phases, namely, transabdominal and transinguinal, regulated by INSL3 and by androgens via the genitofemoral nerve, respectively [2, 3]. The results of experimental studies have confirmed that the synergistic action of testicular INSL3 and androgens contribute to gubernacular development and testicular descent [4, 5]. However, many aspects of impairment of testicular descent are still unclear. In an attempt to clarify these uncertainties, recent studies have hypothesized about the possible role of the appendix testis (AT) in testicular descent noticing a reduced incidence of AT among boys with undescended testes (UDTs) compared to boys without testicular maldescent [6, 7]. According to the best of the author's knowledge, there is no report of the conducted studies that have compared the incidence of AT in congenital UDTs at different localizations.

The purpose of the present study was to determine the potential role of the AT in normal testicular descent by comparing the incidence of AT in two forms of congenital undescended testes: undescended testes located next to the internal inguinal ring and undescended testes located next to the external inguinal ring.

## 2. Patients and Methods

Article is prospective study which included 89 boys with undescended testes who underwent orchidopexy over the period of 4 years at the Clinic of Pediatric Surgery, Clinical Center University of Sarajevo, from January 2011 to December 2014. In patients undergoing orchidopexy due to congenital undescended testis, the age of boys at the time of orchidopexy, the side of orchidopexy, the appearance of testicular appendices, epididymal anomalies, and patency of the processus vaginalis were recorded and evaluated. For the purposes of this analysis, nomenclature of types of undescended testes (UDT) is made based on the recommendations by Radmayr et al. [8]. Retractile testis is the testis that is not in its normal position at the scrotal base but can be located in the inguinal area and manually moved to the base of the scrotum where it will then remain for some minutes without traction or “trapping.” Ectopic testes have somehow left the path of normal descent. They come to reside in the superficial inguinal pouch, the perineum, the femoral canal, and the penopubic area or may be transversly ectopic. True undescended testis is located somewhere along the normal path of descent. Impalpable undescended testis could be “peeping at the internal ring,” when the testis glides from intra-abdominal to a inguinal position or intra-abdominally. In order to determine the clear differentiation of the two groups in our study, Group A indicates UDT close to the internal inguinal ring and Group B indicates UDT near the external inguinal ring (Group A = internal inguinal ring and Group B = external inguinal ring). In relation to the testicular appendices, during a surgical intervention, we have analyzed the presence or absence of testicular appendix (TA), presence or absence of epididymal appendix (EA), presence of multiple EA and presence, or absence of paradidymis or vas aberrans of Haller. If we found testicular appendices during the operation, they were removed.

We have also analyzed the incidence of epididymal anomalies and patency of the processus vaginalis. Testicular appendix (TA) is defined as a vascular nonpedunculated structure attached to the cephalic pole of the testis. Epididymal appendix (EA) is defined as stalked structure attached to the head of the epididymis. Paradidymis or vas aberrans of Haller is defined as structure attached to the lower spermatic cord.

Epididymal anatomy was recorded for all exposed UDT. According to morphological classification by Barthold and Redman [9], epididymal anomalies were defined as anomalies of epididymal fusion consisting of loss of continuity between the testis and the epididymis or long looping epididymis.

The vaginal process (or processus vaginalis) is defined as an embryonic developmental outpouching of the parietal peritoneum. In relation to the processus vaginalis (PV), we have determined two situations: complete obliteration or complete patency of the processus vaginalis. Patients with retractile, ectopic testis or iatrogenic undescended testis were excluded from this study. The frequency of testicular appendices was compared between the two groups. Categorical variables were compared using the Chi-squared test (*χ*2 test). The means of continuous variables were compared using Student's *t*-test, and the data are presented as mean (SD). Statistical level of 95% (*p* < 0.05) was considered as significant for all performed tests.

## 3. Results

**Figure 1:**
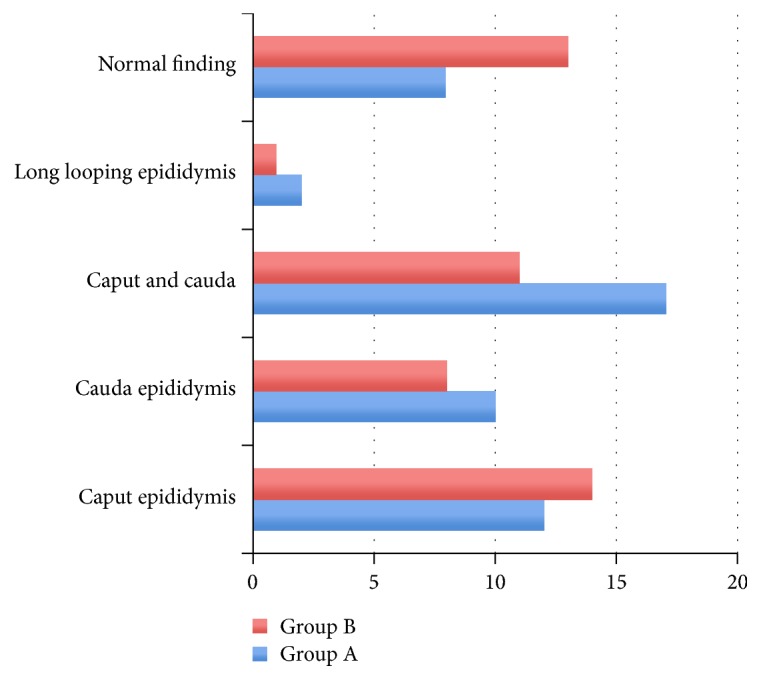
Differentiation of epididymal fusion anomalies in UDTs compared to obliteration.

A total of 89 patients with undescended testes were included in the study. The mean age of boys included in our study was 2.22 years (range from 0.5 to 10.16 years). The majority of patients with UDTs who underwent orchidopexy were below the age of 1 year (51.7%) (*χ*2(3) = 16.80, *p* = 0.0008). The total number of 96 testicular units were assessed. Group A included 42 patients with 49 testicular units and Group B consisted of 47 patients with 47 testicular units. Out of a total of 49 testes in Group A, 44 testes (89.8%) were in the proximal part of the inguinal canal and 5 testes (10.2%) were intra-abdominal in location next to the internal inguinal ring. In our study, there was no statistically significant difference in the gross anatomical appearance of testes in the two groups. Also, there was no statistically significant difference between the two groups regarding the mean age at surgery. Right localization was more represented in the group with testis retention (53.2%) than in the group with cryptorchidism (42.9%), while left and two-sided localization was slightly more represented in the group of patients with cryptorchidism, but without statistically significant difference (*p* > 0.05). Types of epididymal anomalies according to morphological classification by Barthold and Redman [9] were also analyzed. Fusion epididymal anomalies were more frequent in UDTs of Group A (85.7%) than in UDTs of Group B (72.3%), but no statistically significant differences were observed (Figure 1). The incidence of epididymal anomalies was greater in both analyzed groups in cases of complete patency of the processus vaginalis (61.2% of UDTs in Group A and 52% UDTs in Group B) than the incidence associated with complete obliteration of the processus vaginalis (39% of UDTs in Group A and 48% of UDTs in Group B).

Frequency of testicular appendices and patency of the processus vaginalis in relation to the position of the UDTs were analyzed in Group A and in Group B, as shown in Table 1.

The overall incidence of AT in Group A (UDTs localized near to the internal inguinal ring) was 57.1% (28/49), which was significantly lower compared with the incidence of AT in Group B (UDTs localized near to the external inguinal ring) where the AT was seen in 78.7% (37/47) (*p* = 0.020). In UDTs localized near to the internal inguinal ring, only the presence of AT was seen in 23 cases (46.9%), AE in 9 cases (18.4%), and the simultaneous presence of AT and EA in 5 cases (10.2%). The presence of two AE and one AT and paradidymis or vas aberrans of Haller was not found in any UDTs in Group A. In UDTs localized near to the external inguinal ring (Group B), we found only AT in 70.2% cases, AE in 21.3% cases, and the simultaneous presence of the appendix testis and the appendix epididymis in 4 cases (8.5%). Similar to Group A, the presence of two EAs and one AE as well as appendices of paradidymis or vas aberrans of Haller was not found in case included in Group B. No statistically significant difference was found in the presence of EA in UDTs in the analyzed groups. We found that 61.2% UDTs localized near to the internal inguinal ring had patency of the processus vaginalis compared with 53.2% UDTs positioned near to the external inguinal ring. There was no statistically significant difference between the incidence of patency of the processus vaginalis in UDTs of different testicular localization.

## 4. Discussion

Appendices of testis and epididymis, also known as hydatids, are considered to be remnants of the cranial part of the Müllerian duct or Wolffian duct [10]. Appendix testis (AT) is localized at the cranial side of the testis while appendix epididymis (AE) is localized at the head of the epididymis [10]. Embryological development of testicular appendices was the subject of numerous studies. Starting from the known fact that regression of the male Müllerian duct (MD) is mediated by the anti-Müllerian hormone (AMH), numerous studies have shown that apoptosis and cell migration play main roles in this process [11, 12]. Apoptosis of the MD affects all its parts including the cranial tip of the MD. This raised a suspicion about the origin of AT. Various studies have shown that the most cranial part of MD has a special mode of origin and is developing from vestigial nephrostomial pronephric or mesonephric tubules [10, 13, 14]. In males, AT is developed from this part of the MD. Although it was previously considered that the appendix testis (AT) is only vestigial remnant of the paramesonephric (Müllerian) duct with no physiological function in postnatal life, there are published studies that have hypothesized possible functions of AT in humans. Holstein et al. suggested that AT may control amount of serous fluid within the space of tunica vaginalis [15]. At the same time, Posinovec suggested that the surface epithelium, subepithelial capillaries, and lymphatic vessels of AT form functional unit [16]. Ivens confirmed earlier observations that hydatids are organs of resorption with a fluid regulation function within the cavity of the vagina testis [17]. Recently, Samnakay et al. found the expression of androgen and estrogen receptors in the epithelial lining of human ATs [18].

Unlike the vague and insufficiently known function of testicular appendices in prenatal and in part in postnatal life, the clinical relevance of testicular appendices is well known. The possibility of torsion and infarction due to their pedunculate structure have reached the prevailing view that TA should be removed during each surgery for inguinal hernia, hydrocele, or UDT [19].

Although in the last decades there has been a significant increase in findings from many researches, the purpose of testicular descent and factors that enable this process are still not entirely clear [1, 20]. Several hypotheses have been offered to explain testicular descent of which the most popular one is the hypothesis of temperature dependency of spermatogenesis [21]. However, no unified theory explains the cause of testicular descent. There is even greater controversy in explanations of the mechanism of testicular migration process. It is well known that testicular descent is enabled by a combination of growth processes and hormonal influences [22]. Decades of research have led to the discovery of numerous factors involved in testicular descent including gubernaculum testis, the differential growth of abdominal wall, intra-abdominal pressure and temperature, Calcitonin gene related peptide (CGRP), male sex hormones, insulin-like hormone 3 (INSL3), and maternal gonadotrophins. However, many occurrences and events in this process have remained insufficiently explained and some are contradictory.

In order to clarify the process of testicular descent, numerous studies hypothesized about the possible role of AT in normal testicular descent [6, 7, 23]. The role of androgen hormones in the inguinoscrotal phase of the process of testicular descent is well known [24], particularly through mediation in the regression of the cranial suspensory ligament [25]. Since the presence of androgen receptors in ATs has been identified [18, 26], and with the finding that the incidence of ATs is significantly reduced in undescended testes, a hypothesis about a possible role of ATs in testicular descent can be set. In the study conducted by Sahni et al. in order to define the frequency of testicular appendices in 50 neonates, children, and adolescents (aged 1–17 years on medicolegal autopsies), the incidence of TA was 83.3% (88% sessile), while the epididymal appendix was present in 20% (79% stalked) [27].

Results of our study showed significantly decreased incidence of AT in UDTs localized close to the internal inguinal ring compared with those localized close to the external inguinal ring. In contrast to our results, Tostes et al. did not find a significant difference in the number of testicular appendices in UDTs in relation to the testicular position in the patients with undescended testes [23]. However, there are studies that have noted significantly decreased incidence of ATs in UDTs compared with descended testes [6, 7, 27].

The reason for this finding could be explained by the role of the AT in the descent of the testes, either fully or partially which is in accordance with the results of Józsa et al. who concluded that finding of reduced incidence of TAs in UDTs may indicate that AT potentially has an important role in the testicular migration process [7]. Further investigations that focused on the anatomical and functional characteristics of AT during fetal life could offer a clearer understanding of the real role of AT in the process of normal testicular descent. In contrast, no statistically significant difference was found in the presence of EA in UDTs in the analyzed groups. This is fairly consistent with previous studies [6, 27]. Since the presence of two ETs and one AT is a rare anatomic phenomenon [6, 27], our findings confirmed previous observations because neither of the two groups found a common presence of both appendices. UDTs can be associated with a various anatomic anomalies, but most commonly with epididymal anomalies and patency of the process vaginalis [28]. Due to different diagnostic criteria, epididymal and vasal anomalies occur in association with UDT at varying degrees of 32–79% [9, 29–31]. We used morphologic classification proposed by Barthold and Redman [9] to analyze the relationship between the testis and the epididymis. The overall incidence of epididymal anomalies in our study was 78.1%, more frequent in UDTs localized near to the internal inguinal ring (85.7%) than in UDTs localized near to the external inguinal ring (72.3%). These results are fairly consistent with previous findings [9, 29–31]. There was no statistically significant difference between the incidence of epididymal anomalies in UDTs of different testicular localization.

The patency of the processus vaginalis in patients with UDTs ranges from 21.3 to 81.3% [27, 32]. In the present study, we found that 61.2% UDTs positioned near to the internal inguinal ring had patency of the processus vaginalis compared with 53.2% UDTs positioned near to the external inguinal ring. These were consistent with previous findings. There was no statistically significant difference between the incidence of patency of the processus vaginalis in UDTs of different testicular localization. Since studies have shown that the patency of the processus vaginalis at birth is persisted in 80% of children and progressively close during the first year of life under the influence of androgens [33], it is still not sufficiently clear the possible link between the patency of the processus vaginal and the disturbed testicular descent. Further investigations are necessary for clarification of the possible connection of these two phenomena.

Although the present study has certain drawbacks, especially in the absence of a control group of patients, it can offer significant information related to the potential link between testicular appendices and testicular migration process as well as being the basis for further research.

## 5. Conclusion

UDTs positioned close to the internal inguinal ring compared with UDTs positioned close to the external inguinal ring have significantly decreased incidence of TA which may indicate that the presence of ATs may have a role in the testicular migration process.

## Figures and Tables

**Table 1:** Frequency of testicular appendices and patency of the processus vaginalis in relation to the position of the undescended testes.

	Testicular appendices (TA) (localisation)						
	Appendix testis (AT)	Appendix epididymis (AE)	AT and AE	Two AE and one AT	Paradidymis or vas aberrans of Haller	Complete PV patency	Complete PV obliteration
Group A (internal inguinal ring)	23 (46.9%)	9 (18.4%)	5 (10.2%)	0 0%	0 0%	30 (61.2%)	19 (38.8%)
Group B (external inguinal ring)	33 (70.2%)	10 (21.3%)	4 (8.5%)	0 0%	0 0%	25 (53.2%)	22 (46.8%)
