# Corrigendum to “The Role of the Appendix Testis in Normal Testicular Descent: Is There a Connection?”

**DOI:** 10.1155/2019/9296157

**Published:** 2019-04-14

**Authors:** Zlatan Zvizdic, Dragana Zivkovic, Jasmin Sabanovic, Emir Milisic

**Affiliations:** ^1^Clinic of Pediatric Surgery, Clinical Center University of Sarajevo, Sarajevo, Bosnia and Herzegovina; ^2^Medical Faculty, University of Sarajevo, Sarajevo, Bosnia and Herzegovina; ^3^Institute for Child and Youth Health Care of Vojvodina, Faculty of Medicine, University of Novi Sad, Novi Sad, Serbia

In the article titled “The Role of the Appendix Testis in Normal Testicular Descent: Is There a Connection?” [1], the affiliation of the second author was incorrect. The corrected affiliations' list is shown above.
